# Micro-Pattern Guided Adhesion of Osteoblasts on Diamond Surfaces

**DOI:** 10.3390/s90503549

**Published:** 2009-05-13

**Authors:** Bohuslav Rezek, Lenka Michalíková, Egor Ukraintsev, Alexander Kromka, Marie Kalbacova

**Affiliations:** 1 Institute of Physics, Academy of Sciences of the Czech Republic, Cukrovarnicka 10, Prague 6, Czech Republic; E-Mail: ukraintsev@fzu.cz; kromka@fzu.cz; 2 Faculty of Electrical Engineering and Information Technology, Slovak University of Technology, Ilkovičova 3, Bratislava, Slovak Republic; E-Mail: lenka.michalikova@stuba.sk; 3 Institute of Inherited Metabolic Disorders, 1st Faculty of Medicine, Charles University, Ke Karlovu 2, Prague 2, Czech Republic; E-Mail: mkalb@lf1.cuni.cz

**Keywords:** cell adhesion, proteins, diamond, atomic force microscopy, biotechnology, tissue engineering, biosensors, osteoblasts

## Abstract

Microscopic chemical patterning of diamond surfaces by hydrogen and oxygen surface atoms is used for self-assembly of human osteoblastic cells into micro-arrays. The cell adhesion and assembly is further controlled by concentration of cells (2,500-10,000 cells/cm^2^) and fetal bovine serum (0-15%). The cells are characterized by fluorescence microscopy of actin fibers and nuclei. The serum protein adsorption is studied by atomic force microscopy (AFM). The cells are arranged selectively on O-terminated patterns into 30-200 *μ*m wide arrays. Higher cell concentrations allow colonization of unfavorable H-terminated regions due to mutual cell communication. There is no cell selectivity without the proteins in the medium. Based on the AFM, the proteins are present on both H- and O-terminated surfaces. Pronounced differences in their thickness, surface roughness, morphology, and phase images indicate different conformation of the proteins and explain the cell selectivity.

## 1. Introduction

Diamond is not only a famous gemstone but also a promising technological material [1]. Its properties include high hardness, fracture toughness, low friction coefficient, high Young modulus, increased wear resistance and a variety of substrates onto which it can be deposited [2]. Although diamond is considered inert, its surface can be functionalized by various atoms or molecules [3]. This gives rise to striking and unique properties [1]. For instance, electrical conductivity and electron affinity of diamond are strongly influenced by the O- or H-termination of the diamond surface [4, 5]. The differences are mainly caused by the surface dipole of C-H and C-O bonds [6]. O-terminated diamond is highly resistive, whereas H-terminated surface induces p-type surface conductivity even on an undoped diamond [5]. These features can be applied for field-effect transistor (FET) devices [7, 8]. Furthermore, O-terminated surfaces are hydrophilic while H-terminated surfaces are hydrophobic. H-terminated surfaces were thus found less favorable for osteoblastic cell adhesion, spreading and viability compared to O-terminated surfaces [9]. On the other hand, H-terminated diamond surface is an ideal starting point for covalent attachment of biomolecules [10]. Chemical functionalization can also lead to bio-passivation or bio-active properties[11].

This unique combination of the mechanical, chemical, and biocompatible properties [9, 12] with semiconducting properties makes diamond an attractive material for merging solid state and biological systems [13, 14]. For engineered tissue therapies, optimization of implant materials, and cell-based biosensors, characterization of interactions between the cells and surfaces is essential. Cells recognize their surroundings and consequently modify it by a production of appropriate extracellular matrix (ECM) proteins to form the basis for the cell spreading, increased adhesion and expression of differentiated phenotypes [15]. This is a complex and flexible process which is strongly dependent on the cell culture conditions, including the underlying substrate and the pre-adsorbed protein layer. Surface roughness [16] and porosity [17] play significant roles in promoting the cell growth. Hydrophobic and hydrophilic properties of the surfaces influence protein conformations [18, 19] and the cell adsorption and viability [9]. Hence the hydrogen and oxygen-terminated surfaces of diamond are highly relevant for bio-electronics as well as for tissue engineering. So far, the research on the cell-diamond interfaces has been focused mostly on overall homogeneous surface terminations [9, 20, 21].

In this work we show selective adhesion and arrangement of osteoblasts on diamond thin films that are microscopically patterned with H- and O-terminated regions [22, 23]. By controlling the initial cell density and serum concentration in the cell medium we influence cellular colonization of the patterned diamond substrates. Furthermore, we employ atomic force microscopy (AFM) to characterize the structural properties of mediating proteins (fetal bovine serum, a crucial component for the cell growth) adsorbed onto the diamond micro-patterns [19]. The data are used to discuss the selectivity of the cell adhesion on the patterns, i.e. to what degree the cell adhesion and its selectivity is driven by serum adsorption and conformation on H- and O-terminated surfaces or by a direct effect of diamond surface dipoles on the cells. We also provide perspectives for potential bio-electronic applications.

## 2. Experimental Section

Diamond films are grown on (100) oriented silicon substrates (13 mm in diameter, 500 *μ*m thickness, RMS roughness of < 0.6 nm) by microwave plasma process using total gas pressure 50 mbar, substrate temperature 800°C, 1% CH_4_ in H_2_, and total power 2.5 kW. This process results in a growth of continuous, smooth and high quality nanocrystalline diamond (NCD) film [2, 24]. X-ray photocurrent spectroscopy (XPS) detects that the films are 95% pure diamond [25]. The diamond film thickness is 300–400 nm. Average crystal size is 50 nm, RMS roughness at 1×1 *μ*m^2^ area is 15–20 nm as measured by AFM using standard silicon tips of nominal radius < 10 nm. The silicon substrates are coated with NCD film on both sides, silicon is thus hermetically encapsulated in the diamond.

**Figure 1.**
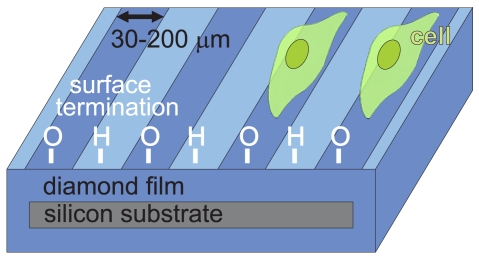
Schematic picture of silicon substrate hermetically coated with diamond layer with stripe-like patterns having hydrogen or oxygen surface termination. Cell adhesion on the O-terminated region is also schematically indicated.

The diamond films were further chemically cleaned in acids (97.5% H_2_SO_4_ + 99% powder KNO_3_) at 200°C for 30 minutes. The surface was then hydrogenated at 800°C for 10 min. Water wetting angle on H-terminated diamond was 80°. NCD films were lithographically processed to generate alternating H- and O-terminated patterns of 30 to 200 *μ*m widths. A positive photoresist ma-P 1215 (micro resist technology GmbH, Germany) was applied. NCD films with the lithographic masks were treated in oxygen radio-frequency plasma (300 W power, 3 min process time) to oxidize the surface and hence to generate the hydrophilic patterns. Then the sample was rinsed in a stripper, de-ionized water and dried. This process removed possible surface contamination [26]. The H/O-termination quality was proved by a scanning electron microscope (SEM; JEOL Superprobe 733). Electronic measurements detected a surface conductivity of 10*^−^*^5^ S/sq on the H-terminated surfaces [27]. Surfaces with O-termination were highly resistive. Water wetting angle on O-terminated diamond was 20°. The NCD samples were sterilized in 70% ethanol for 10 minutes prior to the cell plating. The device concept is schematically shown in Figure 1.

SAOS-2 cells (human osteoblast-like cell line) (DSMZ GmbH), were grown in McCoy's 5A medium (BioConcept) supplemented with heat inactivated fetal bovine serum (FBS; Biowest) of various concentrations (0-15%), penicillin (20 U/mL) and streptomycin (20 *μ*g/mL). We have used osteoblasts because SAOS-2 is a standard immortalized cell line which keeps constant properties during a long period of time. Thus the results can be compared between various series of experiments as well as with reports in the literature. Osteoblasts' response to different materials and in different time periods is well studied, thus we can draw conclusion also from experiments using new materials (diamond) and experimental setups. Osteoblasts are cells generating bone so they are also relevant for osseointegration applications. Cells were plated in the densities of 2,500 and 10,000 cells/cm^2^ using a droplet technique: substrate surface was covered by 100 *μ*L droplet of cell suspension in the appropriately supplemented medium, let to incubate for 2 h (adhesion time), and then 1.4 mL of the medium was added. In the case of 0% FBS, the cells were plated and incubated for 2 h in the medium without the serum. Then the 15% FBS-supplemented medium was added to facilitate further cell cultivation. For comparison, we employed also other cell types: human periodontal ligament fibroblast (HPdLF; Lonza, gift from Dr. Hempel) and human cervical carcinoma (HeLaG; DSMZ GmbH) cells. They were grown in Stromal Cell Basal Medium (Lonza) and DMEM (Gibco), respectively, both supplemented with heat inactivated 15% FBS. After the plating, the cells were cultivated for 48 hours in 5% CO_2_ at 37°C.

An advantage of the applied droplet technique is a precise control of the number of cells applied on the sample. A disadvantage is the slightly non-homogenous distribution of cells over the sample with lower concentration on the edge and higher concentration in the middle of the sample. Therefore, the microscopic images were taken from comparable areas on the samples.

Adhesion and morphology of SAOS-2 cells were characterized by fluorescent staining of actin stress fibers (phalloidin-Alexa 488 - 1:100, Molecular Probes) and nuclei (DAPI - 1:1000, Sigma) according to the protocol in Ref. [28]. The staining was visualized using the E-400 epifluorescence microscope (Nikon); digital images were acquired with a DS-5M-U1 Color Digital Camera (Nikon).

As the adhesion and growth of osteoblastic cells is mediated by proteins, the adsorption, adhesion, and conformation of FBS itself on the H- and O-terminated diamond was also investigated. Polished IIa (100) mono-crystalline diamonds were used as substrates to minimize the contribution from surface morphology of NCD films. The mono-crystalline diamond surface was H- or O-terminated using the same procedures as for the NCD films. A droplet of 15% FBS in the McCoy's 5A medium was applied on the diamond substrates for 10 min. Then the whole samples were immersed in the fluid cell containing the same FBS/McCoy's medium and characterized by AFM (Ntegra, NTMDT). No washing step was applied in between.

AFM measurements were performed in the medium using doped silicon cantilevers (BSMulti75Al) with the typical force constant of 3 N/m, resonance frequency 75 kHz in air (30 kHz in the medium), and nominal tip radius < 10 nm. Surface morphologies were investigated in oscillating-mode AFM (OM-AFM), where the tip-surface interaction is controlled by adjusting the AFM amplitude set-point ratio. Free oscillation amplitude of 60 nm and the set-point ratio of 50% were typically used. The parameters were optimized not to influence the soft FBS layer yet to provide optimal resolution and contrast. A nanoshaving procedure [3, 29] was applied to evaluate the protein layer thickness. First, a region of 2×2 *μ*m^2^ was scanned in contact AFM (C-AFM) and then re-measured across somewhat larger area by OM-AFM. The force applied during C-AFM was approx. 200 nN. The interaction forces in OM-AFM are orders of magnitude lower. The FBS layer thickness was then determined as the difference between average height values across 1 *μ*m^2^ of the FBS layer surface and 1 *μ*m^2^ of the nanoshaved area where FBS was removed. Several regions were probed on each sample to determine the error bar from root-mean- square (RMS) roughness values and statistical errors. Autocorrelation function of the images was calculated to determine typical lateral feature size (Lx).

## 3. Results

**Figure 2.**
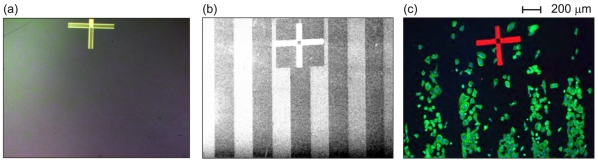
Nanocrystalline diamond film with 200 *μ*m wide H/O-terminated patterns: (a) optical (bright field) image prior to cell plating showing optically transparent and featureless surface, (b) scanning electron microscopy image prior to cell plating where bright stripes correspond to H-termination and dark stripes to O-termination of the diamond surface due to their opposite electron affinity, (c) fluorescent microscopy image of osteoblastic cells cultivated on the substrate. The alignment cross is used for correlation of the surface termination micro-patterns with the cells.

Correlation of oxygen- and hydrogen-terminated micro-patterns on the diamond films with patterns of cell adhesion on such structures is illustrated in Figure 2. Figure 2(a) shows a bright field image of the micro-structured sample in optical microscope before cell seeding. The surface is featureless as the patterns are optically invisible. Figure 2(b) presents a SEM image of the sample, where H- and O-terminated patterns (width of 200 *μ*m) are clearly identified due to their different electronic properties. The bright stripes correspond to the H-terminated NCD surface, having negative electron affinity [5]. The dark stripes represent the O-terminated NCD surface. Fluorescently stained human osteoblasts adherent on 200 *μ*m wide patterned surface are presented in Figure 2(c). By correlating a position of the alignment mark in SEM and fluorescent microscopy pictures, it is evident that the osteoblastic cells preferentially colonize the O-terminated (hydrophilic) patterns.

**Figure 3.**
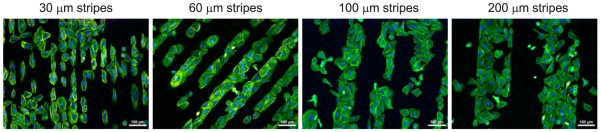
Fluorescent microscopy images of osteoblastic cells (SAOS-2) cultivated in Mc-Coy's medium supplemented with 15% FBS for 2 days on H/O-terminated stripes of different widths (30 *μ*m, 60 *μ*m, 100 *μ*m, and 200 *μ*m) on diamond films. Initial cell concentration was 2,500 cells/cm^2^. The fluorescence shows actin stress fibers (green) and nuclei (blue). Scale bar is 100 *μ*m.

Figure 3 shows that the cells adhere preferentially onto O-terminated stripes independently of the stripe width in the range of 30-200 *μ*m. Two types of cell adhesion patterns are detectable. Cells on the narrow stripes (30 *μ*m - comparable to the cell size) are elongated and form cell-by-cell arrays. On wider stripes (60, 100, and 200 *μ*m - bigger than the cell size) the cells spread and fill the entire width of the stripe. At the micro-pattern borders they form a sharp boundary.

**Figure 4.**
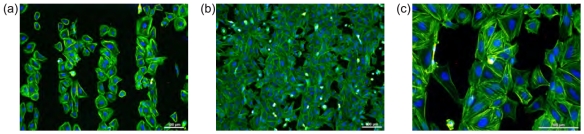
Fluorescent microscopy images of osteoblastic cells (SAOS-2) cultivated for 2 days on 100 *μ*m H/O-terminated stripes on diamond films: (a) low initial cell seeding concentration (2,500 cells/cm^2^), (b) high initial cell seeding concentration (10,000 cells/cm^2^), and (c) cells bridging of unfavorable H-terminated regions. The fluorescence shows actin stress fibers (green) and nuclei (blue). Scale bar is 100 *μ*m.

Osteoblast adhesion onto the NCD surface is affected by the initial cell seeding concentration. Figure 4 illustrates higher selectivity for cell adhesion on the O-terminated surface at lower initial cell seeding density (2,500 cells/cm^2^). There is still some free space for cell spreading and expansion within the hydrophilic region. On the other hand, cells plated at the higher density (10,000 cells/cm^2^) colonize not only hydrophilic areas but also unfavorable hydrophobic regions (Figure 4(b)). Figure 4(c) presents an abnormally long single cell (left image side) as well as clusters of several cells (right image side) that can bridge and colonize the hydrophobic area.

**Figure 5.**
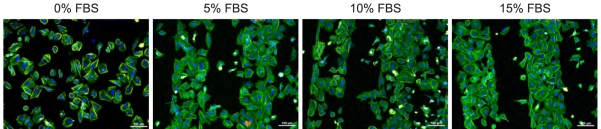
Fluorescent microscopy images of osteoblastic cells (SAOS-2) cultivated for 2 days on 200 *μ*m H/O-terminated stripes on diamond films in McCoy's medium supplemented with different fetal bovine serum (FBS) concentrations (0, 5, 10, and 15%). Note that in the case of 0% FBS, the cells were plated and incubated for 2 h in the medium without the serum, then medium with 15% FBS was added to facilitate further cell cultivation. Scale bar is 100 *μ*m.

Figure 5 demonstrates the influence of different initial FBS concentrations (0, 5, 10, and 15%) in the culture medium on the cell attachment onto the H/O-patterned surface. The range of serum concentrations 5-15% does not significantly affect the cell adhesion pattern. The cells follow the H/O-terminated micro-patterns in the same way as shown in the previous figures. In a sharp contrast, cells plated in FBS-free medium colonize the surface independently of the micro-patterns. The cell selectivity is obviously determined by the FBS proteins.

**Figure 6.**
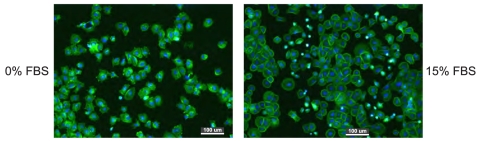
Fluorescent microscopy images of osteoblastic cells (SAOS-2) adsorbed for 2 hours on 200 *μ*m H/O-terminated stripes on diamond films in McCoy's medium supplemented with fetal bovine serum (FBS) concentrations of 0% and 15%. Initial cell concentration was 10000 cells/cm^2^. Scale bar is 100 *μ*m.

Figure 6 shows that the selective cell arrangement on H/O-terminated stripes is detectable already after 2 hours of adsorption in 15% FBS supplemented medium. The cells are again assuming spread shapes on O-terminated surface. There are many more unsettled cells (round shapes and bright dots) on H-terminated surface. The pattern is not yet so well defined as after two days though because the cells had not enough time to spread completely on the surface. Note that no stripes were detected in the control experiment without FBS (run in the same batch). As the cell selectivity is detectable already at quite early stage, not only growth but already the adsorption is strongly influenced by the FBS proteins.

**Figure 7.**
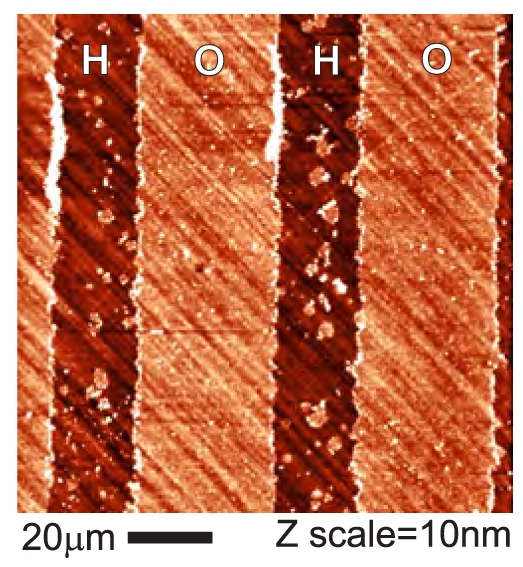
AFM topography image of a fetal bovine serum (FBS) layer on the diamond with stripe-like patterns of H and O surface terminations.

Figure 7 shows OM-AFM topography image of the FBS layer on diamond with stripe-like patterns of hydrogen and oxygen surface terminations. The diagonal lines in the background are due to polishing of the diamond substrate. The roughness of diamond substrate is about 0.6 nm. On this background one can see clear stripes on the O-terminated surface. There are also some small scattered islands of similar thickness on the H-terminated stripes, most likely due to certain degree of non-specific adsorption. When the height of stripes is probed by the nanoshaving method, we find that the layer thickness of the layer adsorbed on O-terminated diamond is 4 ± 2 nm. Even on H-terminated surface (outside of the islands) there is a thin layer of 1.5 ± 2 nm. Hence the FBS layer is present on both types of diamond surfaces, although in the different thickness.

The FBS layer thickness does not depend on the time of adsorption in the range of 10 min to 19 h during continuous in-situ AFM scanning (the topography image remains the same). The protein layer must be thus fully adsorbed within the first 10 min on both types of surfaces. Previous ex-situ measurements indicated that the adsorption of the first monolayer actually occurs within 10 s [19]. The FBS adsorption on diamond is thus very fast compared to typical cell adsorption times (tens of minutes). This adsorption rate is comparable to the protein adsorption on other materials as deduced from quartz crystal microbalance (QCM) experiments [31]. Note also that the thickness on O-terminated diamond is consistently higher than on H-terminated diamond.

**Figure 8.**
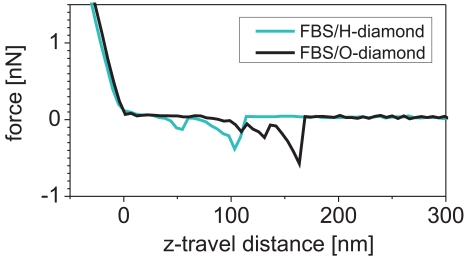
AFM force curves on H- and O-terminated diamond with the adsorbed FBS layer.

Figure 8 shows force curves obtained by force spectroscopy on H/O-terminated diamond patterns after FBS adsorption. The force curves exhibit 500 ± 100 pN interaction between tip and surface on both H-and O-terminated diamond. Similar forces and shapes were found between cantilevers functionalized by bovine serum albumin and glass surfaces after deposition of proteins [30]. Hence the protein molecules from FBS are present also on both H- and O-terminated diamond.

**Figure 9.**
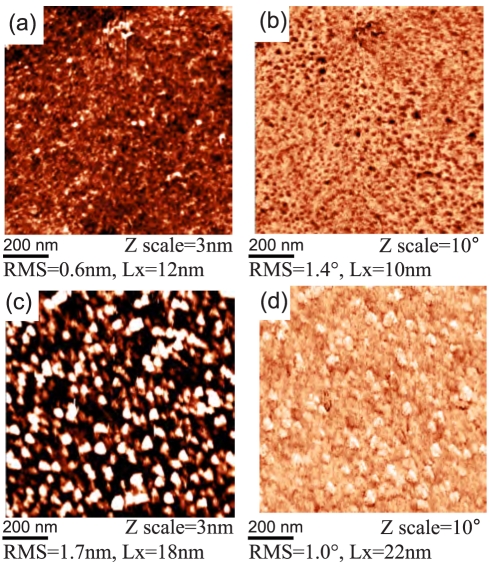
AFM measurements in FBS/McCoy's medium on hydrogen- and oxygen-terminated diamond surfaces with adsorbed FBS layers: topography and phase image on (a-b) FBS/H-terminated diamond (c-d) FBS/O-terminated diamond.

Figure 9 shows the detailed topography and phase images on both types of surfaces. Values of RMS roughness and lateral feature size (Lx) are also given. The FBS layer has a compact form on both H-and O-terminated diamond. The roughness of FBS layer on O-terminated diamond (1.7 nm) is about two times higher compared to H-terminated diamond (0.6 nm). The topographic features are different, with ridge-like shapes around valleys on H-terminated diamond and hillock-like shape on O-terminated diamond. Correspondingly, the feature size is also different, about 10 nm on H-terminated diamond and 20 nm on O-terminated diamond. A pronounced difference is detected also in the AFM phase images. AFM phase image of the adsorbed layer on H-terminated diamond is dominated by dark dots correlated with protrusions in morphology. On O-terminated diamond, brighter spots having darker boundaries are correlated with the hillocks.

**Figure 10.**
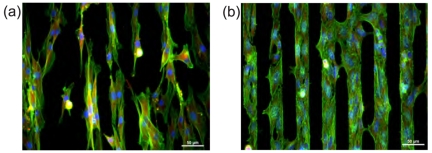
(a) Fibroblasts (HPdLF) and (b) cervical carcinoma (HeLaG) cells cultivated on 30 *μ*m H/O-termination patterns. These cells are aligned on O-terminated regions and form bridges across H-terminated regions. Both images were obtained for the initial cell concentration of 2,500 cells/cm^2^. Scale bar 50 *μ*m.

Figure 10 demonstrates that other cell types are also able to follow the H-/O-termination of micro-patterns. Human fibroblasts (HPdLF) and cervical carcinoma cells (HeLaG) were plated on NCD samples with 30 *μ*m wide stripes and incubated for 48 h. They exhibit different morphologies, yet the same preference to O-terminated diamond surface. Fibroblasts are longer and thinner, forming a lot of fillopodia which helps them to investigate the surrounding in search for suitable area for their growth. They do not exactly fill the entire O-terminated region keeping their typical fibroblastic shape. On the other hand, cervical carcinoma cells fill completely hydrophilic areas and also try to form bridges over the hydrophobic ones. This behavior is not surprising because HeLaG cells are carcinomas, moreover immortalized (like osteosarcomic SAOS-2), thus they do not respect the contact inhibition principle.

## 4. Discussion

In correlation with previous reports on homogeneous surface termination of diamond [9] we find that in case of H/O micro-patterns the cells colonize preferentially hydrophilic (O-terminated) stripes forming confluent arrays with sharp edges separating O- and H- terminated regions. The cells generally did not show any decreased viability, however some of them (preferentially on hydrophobic region) remain rounded for an extended period of time exhibiting poor cell-substratum-compatibility [23, 32]. Evolution of cell morphology on hydrophobic surfaces is slower, but otherwise not remarkably different than that observed for human osteoblasts (hFOB) [32] or SAOS-2 on more hydrophilic surfaces - it is an example of the time-cell-substratum-compatibility-superposition principle.

Also noteworthy is bridging of unfavorable H-terminated regions as illustrated in Figure 4(c). This is obviously enabled by connection to the cells on the O-terminated regions because solitaire cells on the H-terminated regions exhibit bad adhesion and reduced metabolic activity [9, 22]. To reach the optimal status on unfitting surface, cells will communicate with each other, exchanging growth factors and various stimuli as well as produce extracellular matrix (ECM) and thus modify the surface with proteins and proteoglycans underneath to overcome the inhospitable environment. It is known that proteins adsorbed onto the substrate surface do not become permanently immobilized. They will be enzymatically degraded, denatured, they undergo conformational and configuration changes and will even be replaced by other proteins [33]. However, when more cells are able to gently attach to hydrophobic surface in a specific pattern (forming a bridge between two hydrophilic stripes) then these cells may form ECM faster due to support from their proliferating neighbors, thus masking unsuitable properties of the surface. This may be very useful mechanism for bio-electronic applications as it enables to overgrow electrically conductive H-terminated surface when it is surrounded by O-terminated regions at small enough dimensions.

As the cell adsorption is protein mediated, a question arises whether the specific cell adsorption is due to direct effect of diamond surface dipoles on the cells or due to differences in protein adsorption on the micro-patterns. Figure 5 clearly demonstrates that cells plated without proteins (FBS-free medium) do not sense any chemical micro-patterning, whereas cells plated in FBS-supplemented medium clearly follow the hydrophilic patterns. It proves that the cell selectivity is driven by the FBS protein adsorption. Since protein adsorption is much more rapid than the transport of cells to the surface, it is expected that the interaction of host cells with the material is determined by the nature of this adsorbed protein layer. When proteins from FBS attach to H-terminated surface they most likely adopt conformation which causes hiding of the cell-adhering epitopes (e.g. RGD peptide sequence), thus they do not provide optimal condition for the cell adhesion. In the case of FBS-free medium, there is no protein layer to be sensed by the cells during their plating. The cells after short time (2h) are not well spread (looking as star-shapes with many extensions) with some visible contacts to the substrate. Generally, holding mechanism is not known though. After further cultivation (48h) in FBS supplemented medium they adopt their normal shape and grow properly on the spots where they attached (it means everywhere in the case of 0% FBS in the first 2 h and on O-terminated stripes in the case of 15% FBS in first 2h of incubation), because they had enough time to produce their own extracellular matrix and thus to change completely the interface underneath. Diamond H- or O- termination itself is obviously not so critical for the cell selectivity.

AFM study of the protein layers revealed that FBS adsorbs on both types of diamond surfaces. This is in agreement with previous reports that albumin adsorbs on both hydrophilic and hydrophobic surfaces [18]. Here, the adsorbed thickness differs by few nm. It should be noted that FBS layer is a soft matter so there is some uncertainty in determining its thickness by AFM because even in OM-AFM the height may be underestimated [3, 14]. Another influence on the observed step in the height across the nanoshaved region may be a wear of the substrate material. As the flat bulk diamond is very hard compared to proteins and its wear is extremely low, only the FBS layer was penetrated and removed by the nanoshaving forces applied here.

The cell selectivity is thus not determined merely by FBS layer presence. More subtle differences must be considered for explaining the selective adsorption, such as protein denaturation on hydrophobic surfaces [33, 34]. Detailed studies of surface morphology revealed clear differences in surface roughness, morphological features and phase images between the protein layers on H- and O-terminated diamond. Similar difference in topography of proteins on polystyrene substrates were reported in the literature [18]. On hydrophobic surfaces the protein stretches as its hydrophobic core sticks to the surface. On hydrophilic surfaces the protein remains in a rounded form. Hence the most important factor for the cell growth on diamond seems to be the wetting property of the surface rather than any other specific property of the diamond films. This effect is rather general and works also for other cell types.

One has to critically consider that also composition of the adsorbed layers may be different on H- and O-terminated diamond because various proteins (albumin, fibronectin, vitronectin, etc.) from FBS may influence the cell adhesion in different ways. Further experiments are needed to elucidate these details.

## 5. Conclusions

Chemical patterning of diamond films by hydrogen and oxygen surface atoms enables self-assembly of human osteoblastic cell micro-arrays. The cell adhesion and assembly on diamond can be further controlled and optimized by biochemical factors. The cells strongly prefer O-terminated patterns. The best selectivity is achieved for lower initial cell concentrations (2,500 cells/cm^2^), regardless of surface geometry and commonly used protein (FBS) concentrations (5 to 15%). Widths of the patterns affect the shape of adhered cells in the following way: i) good cell spreading with a sharp boundary was observed on broader stripes and ii) elongated cell chains were observed on stripes which were narrower than the cell size. Higher initial concentration of cells enables colonization of less favorable H-terminated surface regions, which are electrically conductive and can be employed in electronic devices. We demonstrated that this effect is general and works also for other cell types. A non-preferential cell adhesion is found when the initial cell adhesion occurs without the serum presence. Hence the cell selectivity is driven by the FBS properties on H- and O-terminated surfaces. AFM detected presence of the FBS layer on both types of surfaces. However, the layer thickness and microscopic morphology are rather different. This may be the reason for the cell selectivity. Further experiments are needed to elucidate details of the selectivity, such as particular composition of the adsorbed layers and so on. Nevertheless, the presented data may already provide valuable information for application of diamond films in tissue engineering, implants, bio-electronics, and biotechnology in general.
